# Technological and Safety Attributes of Lactic Acid Bacteria and Yeasts Isolated from Spontaneously Fermented Greek Wheat Sourdoughs

**DOI:** 10.3390/microorganisms9040671

**Published:** 2021-03-24

**Authors:** Maria K. Syrokou, Sofia Tziompra, Eleni-Efthymia Psychogiou, Sofia-Despoina Mpisti, Spiros Paramithiotis, Loulouda Bosnea, Marios Mataragas, Panagiotis N. Skandamis, Eleftherios H. Drosinos

**Affiliations:** 1 Laboratory of Food Quality Control and Hygiene, Department of Food Science and Human Nutrition, Agricultural University of Athens, 11855 Athens, Greece; syrokoumargia@aua.gr (M.K.S.); stud513026@aua.gr (S.T.); elenhpsycho@yahoo.gr (E.-E.P.); sofiatziompra@outlook.com (S.-D.M.); pskan@aua.gr (P.N.S.); ehd@aua.gr (E.H.D.); 2 Department of Dairy Research, Institute of Technology of Agricultural Products, Hellenic Agricultural Organization “DEMETER”, 45221 Ioannina, Greece; louloudabosnea@gmail.com (L.B.); mmatster@gmail.com (M.M.)

**Keywords:** proteolysis, lipolysis, antimicrobial compounds, plantaricins, *Lactiplantibacillus plantarum*, *Wickerhamomyces anomalus*

## Abstract

The aim of the present study was to assess the technological and safety potential of 207 lactic acid bacteria (LAB) and 195 yeast strains isolated from spontaneously fermented Greek wheat sourdoughs. More accurately, the amylolytic, proteolytic, lipolytic, phytase and amino acid decarboxylase activities, along with the production of exopolysaccharides and antimicrobial compounds by the LAB and yeast isolates, were assessed. A well diffusion assay revealed seven proteolytic LAB and eight yeast strains; hydrolysis of tributyrin was evident only in 11 LAB strains. A further Sodium Dodecyl Sulphate-Polyacrylamide Gel Electrophoresis (SDS-PAGE) indicated partial hydrolysis of gluten. Lipolysis kinetics over 21 days was applied, exhibiting that lipolytic activity ranged from 6.25 to 65.50 AU/mL. Thirteen LAB inhibited *Penicillium olsonii* and *Aspergillus niger* growth and 12 yeast strains inhibited *Pe. chrysogenum* growth. Twenty-one *Lactiplantibacillus plantarum* strains exhibited inhibitory activity against *Listeria monocytogenes*, as well as several sourdough-associated isolates. The structural gene encoding plantaricin 423 was detected in 19 *Lcb. plantarum* strains, while the structural genes encoding plantaricins NC8, PlnE/F, PlnJ/K, and S were detected in two *Lcb. plantarum* strains. None of the microbial strains tested exhibited exopolysaccharide (EPS) production, amino acid decarboxylase, amylolytic or phytase activity. The technological and safety potential of the *Lcb. plantarum* and *Wickerhamomyces anomalus* strains was highlighted, since some of them exhibited proteolytic, lipolytic, antibacterial and antimould activities.

## 1. Introduction

Within the past few years, the ever-increasing consumer demand for “clean label” products has shifted the technological interest of baking industries towards the development of more ecologically friendly methods of preserving foods, such as sourdough fermentation [1]. The incorporation of sourdough into bread making imparts positive effects on all aspects of bread quality, namely technological, sensorial, safety and nutritional attributes. The production of microbial metabolites, which further affect the quality of the end product, is highly dependent on the contribution of lactic acid bacteria (LAB) and yeast strains, which form the sourdough microecosystem [2]. Thus, suitable starter cultures with defined metabolic properties should be carefully selected to assure the reproducibility of the process at industrial level and, at the same time, develop a bakery product with the desired sensorial traits.

As far as the technological properties are concerned, amylase activity in wheat-based sourdoughs is involved in starch hydrolysis, with concomitant liberation of fermentable sugars, namely maltose, glucose and maltodextrines [3]. Despite the fact that α-amylase is regularly absent in flours—meaning its supplementation is necessary in order to increase the presence of fermentable sugars—excessive levels of amylase have been reported as undesirable [4]. However, for the majority of the LAB species derived from wheat-based fermentations, amylase activity has not been considered a common enzymatic property. Apart from amylase activity, amino acids and peptides liberated during proteolysis affect leavened baked goods as both flavor and bioactive compounds [5]. Initial protein degradation to oligopeptides is carried out by cereal proteases, namely aspartic proteases, which are activated under acidic conditions [3]. Further proteolysis is dependent on strain-specific intracellular peptidases of LAB, with the subsequent release of free amino acids [6,7]. The combination of sourdough LAB with mould enzymes has been reported to lead to complete protein hydrolysis, with increased levels of free amino acids [8]. Regarding lipolysis in sourdoughs, only scarce literature is available [2,8]. Although wheat and rye lipids constitute a small part of the corresponding flours, their degradation into flavor precursors, such as aldehydes and alcohols, strongly affects the rheological properties of the final product. As far as homofermentative lactobacilli are concerned, an increase in products from lipid oxidation has been reported [3]. Another enzymatic activity with a nutritional impact, namely phytase activity, results in the degradation of phytic acid present in the cereal grain structure [9,10]. Phytic acid is considered an antinutritional factor due to its ability to create insoluble complexes with essential minerals, namely Ca^2+^, Zn^2+^, Fe^2+^, and Mg^2+^, thus preventing mineral bioavailability [11]. Apart from flour endogenous phytases, several bacterial and yeast isolates have been reported to possess phytase activity, thus contributing to decreased levels of phytate content as well [12,13]. The capacity of sourdough LAB to produce exopolysaccharides (EPS) is another trait with technological significance; this is due to their application as a replacement for commercial hydrocolloids in the bread making process, with subsequent improvements in water absorption of the dough, bread rheology, texture, and shelf life [14]. Apart from technological contributions, EPS from sourdough LAB have been associated with biofilm formation, pathogen exclusion, and prebiotic activity, too. Generally, sourdough LAB have been reported to produce homopolysaccharides (HoPS), while only few a isolates have been associated with heteropolysaccharide (HePS) production [15].

The screening of sourdough microorganisms for antimould and antibacterial activity has been an area of increasing focus, aiming to select appropriate starters that can further improve the shelf life and safety of end products, with respect to consumer demands for less chemical preservatives [16,17]. The capacity of LAB and non-conventional yeast strains to control mould spoilage caused by species of the genera *Aspergillus*, *Penicillium* and *Fusarium* has been thoroughly studied, and is mainly attributed to the synergistic effect of different organic acids, peptides, hydroxyl fatty acids, and phenolic compounds [18,19]. As far as antibacterial capacity is concerned, bacteriocin production has gained much attention the past few years as an alternative biopreservation technique [20]. Although LAB strains producing antibacterial compounds are not intended for extending the shelf life of sourdough fermented cereal foods, metabolic products synthesized during the fermentation process have been reported to enhance the stability of sourdough, thus leading to the production of microbiologically safer products [8]. An additional safety attribute to consider is the inability of starters to form biogenic amines (BAs) during sourdough fermentations [21,22]. In food fermentations, LAB are the main BAs producers through their decarboxylase activity, thus, an increase in BAs accumulation has been reported [23]. In the case of bakery products, although relatively low levels of BAs have been reported [24], the capacity of sourdough microorganisms to form them has not been adequately assessed.

Several authors have studied the properties of yeast and LAB sourdough isolates. However, in the majority of these studies, only a limited set of properties are included, thus offering a restricted interpretation of the capacity of the isolates and the effect they may have on the quality of the final product. The aim of the present study was to evaluate the technological and safety potential of 207 LAB and 195 yeast isolates. More accurately, the amylolytic, proteolytic, lipolytic, phytase and amino acid decarboxylase activities, along with the production of exopolysaccharides and antimicrobial compounds by the LAB and yeast isolates, were assessed.

## 2. Materials and Methods

### 2.1. Microbial Strains and Culture Conditions

A total of 207 lactic acid bacteria and 195 yeast isolates were obtained from thirteen Greek spontaneously fermented wheat sourdoughs [25]. The lactic acid bacteria isolates were identified as follows: *Lactiplantibacillus plantarum* (70 isolates); *Levilactobacillus brevis* (71 isolates); *Companilactobacillus paralimentarius* (30 isolates); *Lvb. zymae* (1 isolate); *Latilactobacillus curvatus* (6 isolates); *Ltb*. *sakei* (12 isolates); *Leuconostoc citreum* (1 isolate); *Ln. mesenteroides* (1 isolate); *Lactococcus lactis* (3 isolates); and *Fructilactobacillus sanfranciscensis* (12 isolates). The yeast isolates were identified as follows: *Saccharomyces cerevisiae* (161 isolates); *Kazachstania humilis* (2 isolates); *Pichia fermentans* (8 isolates); *Pi. membranifaciens* (18 isolates); and *Wickerhamomyces anomalus* (6 isolates). All isolates were stored at −20 °C in a Nutrient broth supplemented with 50% glycerol (Applichem, Darmstadt, Germany). Before experimental use, lactic acid bacteria and yeast isolates were grown twice in de Mann, Rogosa, and Sharpe (MRS) broth, and in Brain Heart Infusion (BHI) broth, and their purity was examined through streaking in MRS agar and BHI agar, respectively. All substrates were from LAB M (Lancashire, UK).

### 2.2. Technological Properties

#### 2.2.1. Production of Exopolysaccharides (EPS)

Screening for EPS production was performed according to Smitinont et al. [26]. More accurately, overnight bacteria cultures were used to inoculate MRS agar containing 2% glucose, fructose, maltose, or sucrose (Applichem). Incubation took place at 30 °C for 3 days. Production of the slimy phenotype was indicative of EPS production.

#### 2.2.2. Amylase Activity

Overnight yeast and bacterial cultures were spot-inoculated on the surface of modified BHI and MRS agar, in which glucose was replaced by 2% soluble starch (Applichem) and incubated at 30 °C for 5 days. Then, the substrate was flooded with Gram’s iodine solution (Sigma-Aldrich, St. Louis, MO, USA). The presence of a clarification halo around each colony was indicative of amylase activity.

#### 2.2.3. Proteolytic Activity

Proteolytic activity was assessed through an agar well diffusion assay. More accurately, in freshly prepared lawns of a medium consisting of 0.5% tryptone (LAB M), 0.25% yeast extract (LAB M), 0.1% glucose (Applichem), 1% gluten (Sigma-Aldrich), and 1.5% agar (LAB M), wells were aseptically punched. Overnight yeast and bacterial cultures were centrifuged (12,000× *g*; 15 min; 4 °C) to obtain cell-free supernatants (CFS). Then, 25 μL of each CFS was added in each well. Incubation took place at 30 °C for 5 days. Then, the substrate was stained with 0.05% (*w*/*v*) Coomassie Brilliant Blue G-250 (Applichem). The presence of a clarification halo around each well was indicative of proteolytic activity.

The strains that exhibited proteolytic activity were subjected to further study. More accurately, the overnight culture was centrifuged (12,000× *g*; 15 min; 4 °C), washed twice with sterile saline, and mixed with a dough made of 10 g wheat flour (T70) and 20 mL tap water. Incubation took place at 30 °C for 24 h. Uninoculated doughs were used as controls. After incubation, albumins, globulins, gliadins, and glutenins fractions were obtained according to Di Cagno et al. [27]. Decomposition of the gluten fractions was assessed through Sodium Dodecyl Sulphate-Polyacrylamide Gel Electrophoresis (SDS-PAGE), in 12% polyacrylamide gel, according to Paramithiotis et al. [28].

#### 2.2.4. Lipolytic Activity

Lipolytic activity was assessed through an agar well diffusion assay. More accurately, the medium consisted of 0.5% peptone (LAB M), 0.3% meat extract (LAB M), 0.5% lecithin (Serva, Heidelberg, Germany), 1% tributyrin (Merck, Darmstadt, Germany), and 1.5% agar, with the addition of 2.5 mM CaCl_2_ (Applichem) and 5 mM MgSO_4_ (Applichem), according to Carrazco-Palafox et al. [29]. Then, wells were aseptically punched. Overnight yeast and bacterial cultures were centrifuged (12,000× *g*; 15 min; 4 °C) to obtain CFS. Twenty-five (25) μL of each CFS were added in each well. Incubation took place at 30 °C for 10 days. The presence of a clarification halo around each well was indicative of lipolytic activity.

Strains that exhibited lipolytic activity were subjected to further study. More accurately, overnight culture was centrifuged (12,000× *g*; 15 min; 4 °C), washed twice with sterile saline, and used to inoculate flasks containing the above medium without agar addition. Incubation took place under shaking (200 rpm) at 30 °C for 21 days. The pH value and total titratable acidity of the samples were determined at days 3, 6, 9, 12, 15, 18 and 21. Uninoculated flasks served as controls. Lipolytic activity was expressed in AU/mL; one arbitrary unit was defined as the amount of enzyme that catalyzed the release of 1 μmol of fatty acids.

#### 2.2.5. Phytase Activity

The protocol described by Anastasio et al. [30] was used to detect phytase activity. In brief, overnight yeasts and bacteria cultures were inoculated into Chalmers broth that was made without neutral red, and with the addition of 1% sodium phytate (Sigma-Aldrich), and incubated at 30 °C for 48 h. Then, a loopful was spotted on the surface of the Chalmers agar that was made without calcium carbonate, and with the addition of 1% hexacalcium phytate (Sigma-Aldrich), and was incubated at 30 °C for 48 h. After incubation, the plates were flooded with 2% (*w/v*) cobalt chloride (Sigma-Aldrich) for 20 min. The presence of a clarification halo around each colony was indicative of phytase activity.

### 2.3. Safety Properties

#### 2.3.1. Production of Antimicrobial Compounds

The well diffusion assay was employed to assess the production of antimould compounds by lactic acid bacteria and yeasts. LAB isolates were further tested for antibacterial activity. Overnight cultures were centrifuged to obtain a CFS that was consequently neutralized and treated with catalase (Sigma-Aldrich). Antibacterial activity was assessed against a mixture of five strains of the foodborne pathogens *Listeria monocytogenes*, *Staphylococcus aureus*, *Escherichia coli* O157:H7, and *Salmonella* serovars. Incubation took place at 37 °C for 24 h. Growth inhibition of the indicator strains around the wells, exceeding 5 mm, was indicative of the presence of antibacterial substances in the cell free supernatant. The strains that exhibited antibacterial activity against the pathogens were further assessed against a mixture of sourdough isolates, namely: *Lcb. plantarum* (LQC 2328, 2330, 2343, 2385, 2462, five isolates); *Lvb. brevis* (LQC 2368, 2429, 2484, 2509, 2518, five isolates); *Cb. paralimentarius* (LQC 2323, 2381, 2399, 2517, 2537, five isolates); *Ltb. sakei* (LQC 2448, 2452, 2456, 2470, 2473, five isolates); *Ltb. curvatus* (LQC 2472, 2475, 2476, 2497, 2498, five isolates); *Fb. sanfranciscensis* (LQC 2402, 2408, 2419, 2425, 2428, five isolates); *Lvb. zymae* (2394, one isolate); *Lc. lactis* (LQC 2375, 2499, 2510, three isolates); *Ln. citreum* (LQC 2508, one isolate); and *Ln. mesenteroides* (LQC 2512, one isolate). Antimould activity was examined against *Penicillium chrysogenum*, *Pe. olsonii*, and *Aspergillus niger* (mouldy bread isolates), as described above, with the exception that incubation took place at 30 °C for 5 days.

The protein nature of the antimicrobial substances was examined by assessing the effect of proteolytic enzymes on antimicrobial activity. More accurately, aliquots of 160 μL CFS were mixed with 40 μL 50 mM phosphate buffer pH 7.5 containing 2 mg mL^−1^ proteinase (Sigma-Aldrich) and 40 μL 50 mM phosphate buffer pH 7.0 containing 2 mg mL^−1^ trypsin (Sigma-Aldrich), and incubated at 37 °C for 1 h. Then, the antimicrobial activity of the CFS was examined as described above.

To determine the effect of pH on the stability of antimicrobial compounds, the pH value of CFS was adjusted to 2, 4, 6, 8 and 10 with 3 M HCl and 3 M NaOH, and incubated at 37 °C for 1 h. To evaluate the thermal stability of the antimicrobial compounds, CFS were treated at 60, 80 and 100 °C for 10 and 30 min, respectively. Untreated CFS were used as controls.

Specific PCR was employed for the detection of the plantaricin structural genes. DNA of the *Lcb. plantarum* strains under study was extracted according to Paramithiotis et al. [2]. The protocol described by Omar et al. [31] was used for the detection of the structural genes encoding plantaricins PlnA, PlnE/F, PlnJ/K, PlnN, NC8, S, and W. Detection of the structural gene of plantaricin 423 was performed in a 20 μL final reaction volume containing 0.2 mM dNTPs, 1.5 mM MgCl_2_, 0.5 μM each primer (413F: TGT GGT AAA CAT TCC TGC TCT G; 413R: CAC TTT CCA TGA CCG AAG TTA GC), and 1 U Taq polymerase (Kappa Biosystems, Boston, MA, USA); the resulting 86 bp amplicon was detected by electrophoresis in a 2% agarose gel.

#### 2.3.2. Biogenic Amine Production

The protocol described by Bover-Cid and Holzapfel [32] was used to assess the amino acid decarboxylase activity of the yeast and bacteria isolates. More accurately, overnight yeast and bacteria cultures were spot-inoculated on the surface of a medium consisting, per liter, of 5 g yeast extract, 5 g tryptone, 5 g meat extract, 5 g glucose, 2.5 g sodium chloride, 2 g ammonium citrate, 1 g Tween 80, 0.2 g MgSO_4_, 0.05 g MnSO_4_, 0.04 g FeSO_4_, 0.01 g thiamine, 2 g K_2_PO_4_, 0.1 g CaCO_3_, 0.05 g pyridoxal-5-phosphate, 0.06 g bromocresol purple, and 20 g agar, and supplemented with 10 g lysine, tyrosine, ornithine, or histidine, pH 5.3. Incubation took place at 30 °C for 48 h. Occurrence of color change around the colonies, from yellow to purple, was indicative of decarboxylation of the respective amino acid. All chemicals were from Sigma-Aldrich.

### 2.4. Statistical Analysis

One-way ANOVA was employed to assess the statistical significance of the differences observed in the lipolysis kinetics between the microbial strains.

## 3. Results

### 3.1. Assessment of Technological Properties

#### 3.1.1. Proteolytic Activity

**Figure 1:**
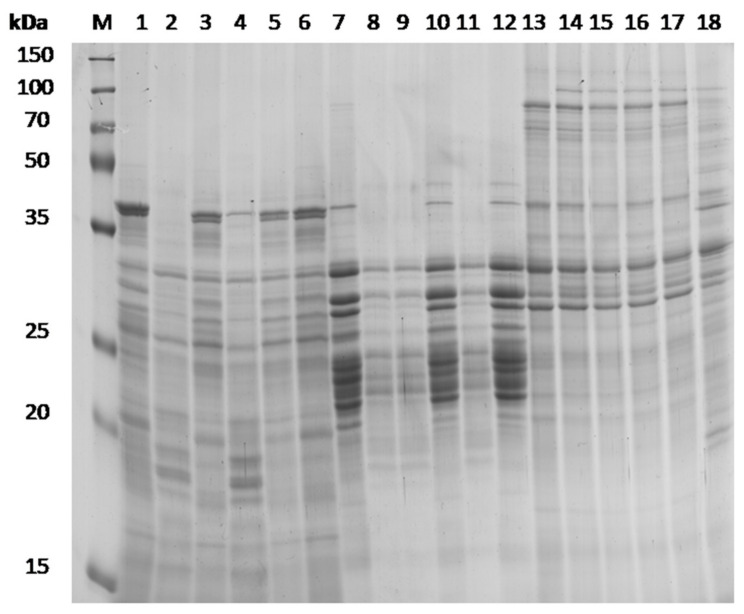
Representative SDS-PAGE analysis of gluten fractions extracted from a mixture of wheat flour and water (1:2), incubated for 24 h at 30 °C, after inoculation with different LAB and yeast strains, which previously displayed positive proteolytic activity. M, protein marker; Lane 1, albumin fraction extracted from dough without inoculum (control); Lanes 2–6, albumin fractions extracted from doughs inoculated with microbial strains LQC 2320, 2474, 2520, 2469, and 10343, respectively; Lane 7, gliadin fraction extracted from dough without inoculum (control); Lanes 8–12, gliadin fractions extracted from doughs inoculated with microbial strains LQC 2320, 2520, 10343, 2469, and 2474, respectively; Lane 13, glutenin fraction extracted from dough without inoculum (control); Lanes 14–18, glutenin fractions extracted from doughs inoculated with microbial strains LQC 2320, 2469, 2474, 10343, and 2520, respectively.

The agar well diffusion assay revealed that, of the 207 LAB and 195 yeast strains initially screened for proteolytic activity, seven and eight strains, respectively, were able to hydrolyze gluten. A clear halo around the wells was present for these 15 strains, indicating the presence of proteolytic capacity. Regarding LAB isolates, four belonged to *Lcb. plantarum* (LQC 2320, 2372, 2464, 2520) and three to *Lvb. brevis* (LQC 2469, 2474, 2493). In the case of yeasts, four isolates were assigned to *S. cerevisiae* (LQC 10343, 10378, 10398, 10402), three to *W. anomalus* (LQC 10346, 10353, 10360), and one to *Pi. fermentans* (LQC 10349). The proteolytic capacity of these 15 microbial strains was further evaluated with SDS-PAGE analysis. The SDS-PAGE confirmed the gluten degrading potential of three LAB strains, namely LQC 2320, 2469, and 2520. More accurately, sourdough fermentation with the specific monocultures of LAB strains resulted in significant hydrolysis of albumins and gliadins (Figure 1). Hydrolysis was partial or absent towards glutenins. Compared to the albumin control, sourdough fermentation with LAB strains LQC 2520 and 2320 resulted in complete and partial hydrolysis, respectively, of the albumin band with molecular weight (Mw) of ca. 40 kDa. In addition, the band intensity of albumins with Mw ranging between 25–35 kDa decreased, while the disappearance of the albumin bands in the range of 20–25 kDa, due to extensive hydrolysis, was also evident. The appearance of additional albumin bands of lower Mw, ranging between 15–20 kDa and belonging to doughs fermented by LAB strains LQC 2520 and 2320, respectively, was also shown in the electrophoretic analysis.

In the case of gliadins, LAB strains LQC 2320, 2520, and 2469 revealed similar profiles of proteolysis. Compared to the control, complete hydrolysis of gliadin bands corresponding to ca. 40 and 20 kDa was detected in SDS-PAGE. After sourdough fermentation with each of the aforementioned LAB strains, the intensity of gliadin bands with Mw between 20–35 was significantly decreased. As far as the third gluten fraction was concerned, glutenins extracted from dough previously inoculated with LAB strain LQC 2520 revealed additional bands between 35–70, and below 25 kDa. The rest of the glutenins extracted were not affected by LAB fermentation.

#### 3.1.2. Lipolytic Activity

The agar well diffusion assay revealed that, of the 207 LAB strains tested, 11 were found to be lipase positive, among which six were identified as *Lcb. plantarum* (LQC 2320, 2321, 2385, 2397, 2516, and 2520), four as *Lvb. brevis* (LQC 2374, 2411, 2416, and 2432), and one as *Cb. paralimentarius* (LQC 2410). None of the 195 yeast strains tested were able to hydrolyze tributyrin. A further lipolysis kinetics over 21 days was performed, with lipase activity expressed in AU/mL, as shown in Table 1. Data obtained revealed that *Lcb. plantarum* LQC 2321 and 2397 and *Lvb. brevis* LQC 2416 presented the highest enzyme activities of 62.75, 65.40, and 55.75, respectively, at the day 18 of incubation, with lipase activities remaining almost constant until day 21 of incubation.

Similarly, *Lvb. brevis* LQC 2374, *Cb. paralimentarius* LQC 2410, and *Lcb. plantarum* 2516 exhibited the lowest lipase activities until day 21 of incubation (6.25, 17.00 and 14.50 AU/mL, respectively). The other LAB strains exhibited moderate lipase activities, ranging from 22.50 to 47.75, until day 21 of incubation.

None of the microbial strains tested exhibited EPS production, amylolytic, or phytase activity.

### 3.2. Assessment of Safety Properties

#### 3.2.1. Antimicrobial Activity

Among the 207 bacterial and 195 yeast strains initially evaluated against *Pe. chrysogenum*, *Pe. olsonii*, and *A. niger* by the agar well diffusion assay, 13 and 12, respectively, were able to inhibit mould growth. In the case of LAB, the following showed extensive inhibition against *Pe. olsonii* and *A. niger*: two *Lcb. plantarum* (LQC 2321 and 2327), two *Lvb. brevis* (LQC 2523 and 2529), two *Ltb. sakei* (LQC 2448 and 2454), one strain identified as *Fb. sanfranciscensis* (LQC 2419), *Cb. paralimentarius* (LQC 2392), *Ltb. curvatus* (LQC 2476), *Lvb. zymae* (LQC 2394), *Lc. lactis* (LQC 2499), *Ln. mesenteroides* (LQC 2512), and *Ln. citreum* (LQC 2508). As far as yeasts were concerned, seven *S. cerevisiae* (LQC 10286, 10298, 10307, 10393, 10404, 10421, and 10469), three *W. anomalus* (LQC 10346, 10353, and 10360), and two *Pi. fermentans* (LQC 10355 and 10356) presented inhibition against *Pe. chrysogenum*. LAB strains exhibited no inhibitory activity against *Pe. chrysogenum*, while *Pe. olsonii* and *A. niger* showed great resistance against all yeast strains.

The antibacterial capacity of LAB strains was also tested against foodborne pathogens *L. monocytogenes*, *S. aureus*, *E. coli* O157:H7, and *Salmonella* serovars. Twenty-one *Lcb. plantarum* strains (LQC 2320, 2384, 2422, 2441, 2442, 2443, 2444, 2445, 2446, 2447, 2485, 2486, 2487, 2488, 2489, 2490, 2491, 2492, 2506, 2516, and 2520) exhibited inhibitory properties against a mixture of *L. monocytogenes* 4b strains. No inhibitory potential of LAB strains evaluated against *S. aureus*, *E. coli* O157:H7, and *Salmonella* serovars was observed. These 21 *Lcb. plantarum* strains were further shown to inhibit sourdough isolates of *Lcb. plantarum*, *Lvb. brevis*, *Cb. paralimentarius, Ltb. sakei*, *Ltb. curvatus*, *Fb. sanfranciscensis*, and *Lc. lactis*. No inhibitory activity against *Lvb. zymae*, *Ln. citreum*, and *Ln. mesenteroides* was observed.

The structural gene encoding plantaricin 423 was detected in nineteen *Lcb. plantarum* strains. On the contrary, the structural genes encoding plantaricins NC8, PlnE/F, PlnJ/K, and S were detected in strains LQC 2320 and 2520.

None of the LAB and yeast isolates presented production of biogenic amines.

#### 3.2.2. Effect of Proteolytic Enzymes, pH, and Temperature on the Stability of Antimould and Antibacterial Compounds Produced by Sourdough LAB and Yeasts

The CFS obtained from LAB and yeast strains, which previously demonstrated antimicrobial activity, were further assessed for their stability upon enzymatic, pH, and thermal treatment. The antibacterial compounds towards *L. monocytogenes* produced by 21 *Lcb. plantarum* strains were of proteinaceous nature, as the inhibitory action was abolished after treatment with at least one of the proteolytic enzymes employed, namely proteinase and trypsin. As far as the effect of thermal and pH treatment was concerned, these antibacterial substances demonstrated a highly thermostable and pH tolerant profile, since activity was retained after any temperature or pH treatment was applied. The antimould compounds produced by both LAB and yeast strains were non proteinaceous, as their activity remained even after enzymatic treatment. Regarding thermal stability, the CFS from all microbial strains tested completely lost their antimould capacity after thermal treatment. On the contrary, antimould compounds from both LAB and yeast strains exhibited a pH tolerant profile at all different pH values, ranging from 2 to 10.

## 4. Discussion

In the past few years, increasing demand for large-scale and controllable bread production has driven both scientific and industrial attention towards a more careful selection of sourdough starters. Desired technological and safety properties of candidate starters are those determining the potential use of sourdough microbial strains in the production of baked goods.

Proteolysis during sourdough fermentation has been considered a key process for determining the dough rheology and overall quality of the final product. Thus, the presence of proteolytic activity is a selection criterium of paramount importance among candidate sourdough starters [33,34]. Primary gluten hydrolysis is dependent on cereal proteases, while secondary proteolysis is carried out by strain-specific peptidases of sourdough LAB [35]. Except for LAB acidification, increased levels of thiol groups in the gluten proteins, produced via the glutathione reductase activity of heterofermentative lactobacilli, contribute to the depolymerization of glutenin macropolymer, thus increasing their susceptibility to enzymatic degradation [8]. In the present study, the initial test revealed seven proteolytic LAB strains and eight proteolytic yeast strains. However, the SDS-PAGE confirmed the proteolytic capacity for only three LAB strains. Similar results are often reported and may be attributed to insufficient incubation time [36,37,38]. Lancetti et al. [34] have already reported the proteolytic capacity of *Lcb. plantarum* ES137 and *Pd. acidilactici* ES22, previously isolated from Argentinian grains, by applying SDS-PAGE analysis. More accurately, sourdoughs inoculated with *Lcb. plantarum* ES137 and *Pd. acidilactici* ES22 presented a different protein profile in electrophoretic gel, compared to the controls, which is characterized by a decreased band number and intensity. Another study reported that the application of SDS-PAGE for characterization of the protein content of sourdoughs, inoculated with a mixed culture of *Lcb. plantarum* and yeast, revealed extensive hydrolysis of higher molecular weight protein bands, with the concomitant appearance of additional bands of lower molecular weight [39]. In the case of yeasts, decreased proteolytic activity has been reported by the majority of researchers [33]. Generally, yeasted doughs are characterized by decreased levels of amino acids compared to doughs fermented with LAB [40].

Lipolysis has been extensively studied in fermented dairy, non-dairy and meat products [38,41,42]. Despite the fact that a certain level of lipolysis is a desirable attribute of microbial strains, in terms of synthesis of flavor precursors, only a few pieces of scientific literature have reported the microbial screening for lipolytic activity of sourdough microorganisms [2]. At first, lipases hydrolyze triacylglycerols into free fatty acids [43]. Then, unsatturated fatty acids, namely linoleic acid, the major component of cereal lipids, are further degraded into peroxides through either autoxidation during flour storage or cereal lipoxygenase activity during dough mixing [44]. Finally, degradation of peroxides into aldehydes takes place, with the latter being further reduced to alcohols via heterofermentative lactobacilli during sourdough fermentation. The agar well diffusion assay applied in the present study revealed 11 lipase positive LAB strains of the 207 tested; the lipolysis kinetics further applied over 21 days revealed that *Lcb. plantarum* LQC 2321 and LQC 2397 and *Lvb. brevis* LQC 2416 presented the highest lipolytic activity on day 21 of incubation. None of the yeasts tested exhibited lipolytic activity. To our knowledge, no previous study has reported the presence of lipolytic microbial strains isolated from spontaneously fermented sourdoughs; except for Paramithiotis et al. [28], who reported the presence of *Yarrowia lipolytica* in Greek sourdough microbiota. *Y. lipolytica* strains are known for their strong lipolytic and proteolytic activity [45]. Regarding LAB, they are considered as weak lipolytic compared to other bacterial species, such as *Pseudomonas* spp., *Acinetobacter* spp., and *Flavonobacterium* spp. Indeed, the absence of lipolytic activity among strains of *Lcb. plantarum* and *Lvb. brevis* was reported by Kamiloğlu et al. [46] and Ebadi Nehzad et al. [47]. However, another study revealed that, of the 137 bacterial strains initially screened for proteolytic and lipolytic activity, seven exhibited both [41]. Among them, two belonged to *Lcb. plantarum*. Zymography was further applied to isolate the proteins responsible for exhibiting lipolytic activity; however, no lipolytic proteins were detected in the case of *Lcb. plantarum.* Regarding *Cb. paralimentarius*, this is the first study to report its lipolytic activity.

Sourdough fermented with antimould LAB and yeast strains has been the epicenter of intensive study over the past few years; it is an alternative biopreservation approach in line with consumer demand for clean label products [48]. The most common moulds spoiling bread products belong to the genera *Aspergillus*, *Penicillium*, *Fusarium*, and *Cladosporium*, and their presence constitutes a major concern for baking industries due to the potential of mycotoxin production, with concomitant long term health risks [1]. Antimould metabolites synthesized by sourdough LAB and yeast strains are responsible for biopreservation effects. In the present study, 13 LAB inhibited *Pe. olsonii* and *A. niger* growth and 12 yeast strains inhibited *Pe. chrysogenum* growth. The antimould activity of the majority of LAB species tested in the present study has been previously reported against numerous moulds which commonly spoil bread. More accurately, Fraberger et al. [1] reported the strain-dependent antimould potential of wheat and rye sourdough-derived lactobacilli; the majority of the isolates belonging to *Lcb. plantarum* presented wide antimould activity against all mould strains tested. In the case of *Lvb. brevis*., isolate S4.5 strongly inhibited *Pe. roqueforti*, while the mould remained resistant against *Lvb. brevis* S13.18, S14.3, and S6.13. Isolates belonging to *Fb. sanfranciscensis*, *Ltb. sakei* S4.19, and *Ltb. curvatus* S4.14, S5.22, S6.15 exhibited very strong inhibition against *F. graminearum*. In addition, *Cb. paralimentarius* S7.5 exhibited very strong inhibitory activity against *A. fumigatus* and *F. graminearum*. An additional study by Manini et al. [49] reported the antimould activity of wheat bran sourdough-originating *Lcb. plantarum*, *Lvb. brevis*, *Ltb. curvatus*, and *Ltb. sakei* against *A. oryzae* and *A. niger*. *Ln. mesenteroides* and *Ln. citreum* strains were also able to inhibit both *Aspergillus* species. As far as *Lc. lactis* was concerned, strain CH179 derived from chia flour fermentation presented inhibitory activity against *A. niger* and *Pe. roqueforti* [50]. In the case of *Lvb. zymae*, the antimould activity of a sourdough-derived strain has not been reported so far, while few studies have documented *Lvb. zymae* as inactive against mould growth [51]. Regarding yeasts, the literature is focused on their leavening capacity, flavor development, and mutualistic interaction with LAB, while only few scientific data have reported their role as antimould agents [18,52]. More accurately, Jin et al. [53] reported the absence of antimould activity of sourdough-derived *S. cerevisiae* against *A. flavus*. In addition, its combination with antimould *Pd. pentosaceus*, and their application as starters in sourdough fermentations, presented the greatest inhibitory effect against *A. flavus*. Regarding *W. anomalus*, a strong inhibitory activity against a range of moulds has been reported when used as starter in dough fermentation. Sourdough fermented with a combination of *W. anomalus* with *Lcb. plantarum* exhibited slightly decreased antimould activity; however, sourdough bread delayed mould growth until 28 days of storage. The antimould peptides and ethyl acetate synthesized by *Lcb. plantarum* and *W. anomalus*, respectively, were responsible for the extension of the mould-free shelf life of bread. With respect to *Pi. fermentans*, no previous study has reported the antimould activity of this sourdough originating yeast. However, the antimould potential of coffee fruit-derived strain LPBYB13 was reported against an ochratoxigenic strain of *A. westerdijkiae* on agar tests, and a further inhibition of ochratoxin A production in coffee beans was performed [54].

The antibacterial potential of LAB is a significant criterium for the selection of more competitive starters in sourdough fermentations, and could determine the microbiological stability and safety of the baked products. Despite the fact that the antibacterial compounds produced by sourdough LAB, namely bacteriocins, bacteriocin-like inhibitory substances (BLIS), and antibiotics, do not extend the mould-free shelf life of the end products, they positively affect their microbiological safety by counteracting food contamination during processing [55]. *L. monocytogenes*, *S. aureus*, *E. coli*, and *Salmonella* serovars are the most common foodborne pathogens. In the present study, of 207 LAB isolates evaluated against food borne pathogens, 21 *Lcb. plantarum* strains exhibited inhibitory activity against a mixture of *L. monocytogenes* 4b strains. These 21 *Lb. plantarum* strains further inhibited several sourdough-associated LAB. In agreement with the present data, hull-less barley sourdough-originating *Lcb. plantarum* SAB15 exhibited a very strong inhibitory potential against *B. subtillis*, *B. cereus*, and *E. coli*, based on agar diffusion assay [9]. In addition, Demirbas et al. [56] reported the antibacterial activity of Turkish wheat sourdough-derived *Lcb. plantarum* ED10 against *B. cereus*, *S. aureus*, *Y. enterocolitica*, and *E. coli*. The DNA sequence of the structural genes encoding eight plantaricins, namely NC8, PlnA, PlnE/F, PlnJ/K, PlnN, W, S, and 423, has been verified so far. The latter is plasmid-encoded, while the rest are chromosomally-encoded [57,58]. Their characteristics and applications have been also reviewed for the majority of them [20,59]. To our knowledge, this is the first study reporting the presence of plantaricins NC8, PlnE/F, PlnJ/K, S, and 423 from sourdough-derived *Lcb. plantarum* isolates. Previous studies have reported only the production of plantaricin ST31 [60] and plantaricin A [61] from sourdough-derived strains of *Lcb. plantarum*.

The presence of phytase and amylase positive sourdough microorganisms is of paramount importance from a technological perspective. During sourdough fermentation, phytases dephosphorylate phytic acid into myo-inositol and phosphoric acid, leading to increased mineral bioavailability and further improvement of the nutritional characteristics of the final product. Regarding amylases, they are responsible for starch hydrolysis, with concomitant formation of fermentable sugars. In the present study, no phytase and amylase positive LAB and yeast strains were detected. In line with the present data, Paramithiotis et al. [2] detected no amylolytic activities of sourdough LAB and yeast strains tested. On the other hand, several studies have reported the presence of sourdough-derived microbial strains with phytase activity [62,63]. Another property with technological impact, however, not detected in the present study, was EPS production by LAB strains. On the contrary, previous studies by Milanović et al. [64] and Manini et al. [49] reported the presence of EPS producing LAB strains, derived from cereal based substrates and wheat bran sourdoughs, respectively. A last safety attribute assessed in the present study, but what was not detected in either LAB or yeast strains tested was the ability to form BAs. Within permissible limits, BAs exert no adverse health effects on consumers; however, when accumulated in food products, they can possibly present a health risk. Compared to other fermented foods, BA production in sourdough fermentations is of less concern, with BA levels ranging far below those posing health risks [24]. In agreement with the previous statement, Bartkiene et al. [65] reported on BA production in the solid-state fermentation of flaxseed; however, the levels were relatively low to pose health issues.

## 5. Conclusions

The results obtained in the present study clearly reveal the technological and safety potential of several bacterial and yeast strains. Two *Lcb. plantarum* strains, LQC 2320 and 2520, exhibited proteolytic, lipolytic and antibacterial capacity. The presence of structural genes encoding plantaricins NC8, PlnE/F, PlnJ/K, and S was also detected in both LAB strains. In the case of yeasts, three *W. anomalus* strains, LQC 10343, 10353, and 10360, exhibited both proteolytic and antimould potential. These properties deserve further research due to the impact they may have on the quality of sourdough bread.

## Figures and Tables

**Table 1:** Lipolysis kinetics of 11 bacterial strains during 21 days of incubation, at 30 °C.

Sourdough LAB Strains	Days						
	3	6	9	12	15	18	21
*Lcb. plantarum* LQC 2320	8.75 ± 1.06 ^e,E^	23.75 ± 1.06 ^g,G^	34.00 ± 0.00 ^g,C^	35.50 ± 0.71 ^d,D^	36.00 ± 0.00 ^e,D,E^	37.50 ± 0.71 ^e,E^	37.25 ± 0.35 ^e,E^
*Lcb. plantarum* LQC 2321	2.50 ± 0.71 ^ab,AB^	11.50 ± 0.71 ^e,E^	42.00 ± 0.00 ^h,C^	52.50 ± 0.71 ^g,G^	59.50 ± 0.71 ^i,E^	62.75 ± 1.06 ^i,F^	63.00 ± 0.00 ^i,F^
*Lvb. brevis* LQC 2374	3.00 ± 0.00 ^a,ABC^	3.25 ± 0.35 ^ab,A^	3.50 ± 0.00 ^abc,AB^	4.00 ± 0.00 ^bc,A^	4.25 ± 0.35 ^c,B^	6.50 ± 0.71 ^d,C^	6.25 ± 0.35 ^d,C^
*Lcb. plantarum* LQC 2385	1.50 ± 0.71 ^a,A^	5.20 ± 0.42 ^b,B^	9.55 ± 0.78 ^bc,C^	14.50 ± 0.71 ^b,B^	19.50 ± 0.71 ^d,E^	22.50 ± 0.71 ^d,F^	22.50 ± 0.71 ^d,F^
*Lcb. plantarum* LQC 2397	5.50 ± 0.71 ^d,D^	11.25 ± 1.06 ^e,E^	49.00 ± 1.41 ^i,C^	57.50 ± 0.71 ^h,H^	61.50 ± 0.71 ^k,E^	65.40 ± 0.85 ^j,F^	65.50 ± 0.71 ^j,F^
*Cb. paralimentarius* LQC 2410	3.00 ± 0.00 ^abc,ABC^	7.50 ± 0.71 ^cd,CD^	8.45 ± 0.78 ^b,B^	14.25 ± 1.06 ^b,B^	16.00 ± 0.00 ^c,D^	17.25 ± 0.35 ^c,D^	17.00 ± 0.00^c,D^
*Lvb. brevis* LQC 2411	9.00 ± 0.71 ^e,E^	13.25 ± 1.06 ^f,F^	19.75 ± 0.35 ^e,C^	34.50 ± 0.71 ^d,D^	37.50 ± 0.71 ^f,E^	48.60 ± 0.85 ^g,F^	47.75 ± 1.06 ^g,F^
*Lvb. brevis* LQC 2416	5.50 ± 0.71 ^d,D^	10.50 ± 0.71 ^e,E^	42.75 ± 0.35 ^h,C^	49.75 ± 0.35 ^f,F^	55.25 ± 0.35 ^h,E^	55.75 ± 0.35 ^h,E^	55.75 ± 0.35 ^h,E^
*Lvb. brevis* LQC 2432	1.75 ± 0.06 ^a,A^	13.50 ± 0.71 ^f,F^	23.75 ± 1.06 ^f, C^	41.50 ± 0.71 ^e,E^	41.75 ± 0.35 ^g,D^	45.50 ± 0.71 ^f,E^	46.00 ± 0.71 ^f,E^
*Lcb. plantarum* LQC 2516	3.50 ± 0.71 ^bc,BC^	6.25 ± 0.35 ^bc,BC^	10.50 ± 0.71 ^c,C^	14.50 ± 0.71 ^b,B^	14.50 ± 0.71 ^b,D^	14.75 ± 0.35 ^b,D^	14.50 ± 0.71 ^b,D^
*Lcb. plantarum* LQC 2520	4.50 ± 0.71 ^cd,CD^	8.00 ± 0.71 ^d,D^	15.50 ± 0.71 ^d,C^	17.25 ± 0.35 ^c,C^	18.50 ± 0.71 ^d,D^	22.50 ± 0.71 ^d,E^	22.75 ± 0.35 ^d,E^

The lipase activity is expressed in AU/mL. *Cb*.: *Companilactobacillus*; *Lcb*.: *Lactiplantibacillus*; *Lvb*.: *Levilactobacillus*. Statistically significant inter-species and intra-species differences are expressed with different letters—a–j and A–F, respectively.

## Data Availability

The data presented in this study are available in the manuscript.
